# Design of a Multiepitope Vaccine against Chicken Anemia Virus Disease

**DOI:** 10.3390/v14071456

**Published:** 2022-06-30

**Authors:** Abiodun Joseph Fatoba, Victoria T. Adeleke, Leah Maharaj, Moses Okpeku, Adebayo A. Adeniyi, Matthew A. Adeleke

**Affiliations:** 1 Discipline of Genetics, School of Life Sciences, University of KwaZulu-Natal, Westville Campus, P/Bag X54001, Durban 4000, South Africa; leahmaharaj@gmail.com (L.M.); OkpekuM@ukzn.ac.za (M.O.); 2 Department of Chemical Engineering, Mangosuthu University of Technology, Umlazi, Durban 4031, South Africa; vickteni2006@yahoo.com; 3 Department of Industrial Chemistry, Federal University, Oye-Ekiti P.O. Box 370111, Nigeria; adebayo.adeniyi@fuoye.edu.ng; 4 Department of Chemistry, University of the Free State, P.O. Box 339, Bloemfontein 9300, South Africa

**Keywords:** chicken anemia virus, disease, immunoinformatics, immune response, multiepitope, viral proteins

## Abstract

Chicken anemia virus (CAV) causes severe clinical and sub-clinical infection in poultry globally and thus leads to economic losses. The drawbacks of the commercially available vaccines against CAV disease signal the need for a novel, safe, and effective vaccine design. In this study, a multiepitope vaccine (MEV) consisting of T-cell and B-cell epitopes from CAV viral proteins (VP1 and VP2) was computationally constructed with the help of linkers and adjuvant. The 3D model of the MEV construct was refined and validated by different online bioinformatics tools. Molecular docking showed stable interaction of the MEV construct with TLR3, and this was confirmed by Molecular Dynamics Simulation. Codon optimization and in silico cloning of the vaccine in pET-28a (+) vector also showed its potential expression in the *E. coli* K12 system. The immune simulation also indicated the ability of this vaccine to induce an effective immune response against this virus. Although the vaccine in this study was computationally constructed and still requires further in vivo study to confirm its effectiveness, this study marks a very important step towards designing a potential vaccine against CAV disease.

## 1. Introduction

Chicken anemia virus (CAV) is a global challenge to the poultry industry as it causes severe anemia, hemorrhages, and immunosuppression in young chickens, leading to considerable economic losses [1,2]. Young chicks less than 3 weeks of age with maternally derived antibodies (MDA) are usually protected from severe clinical symptoms caused by the vertical transmission of this virus [1]. However, sub-clinical infections (such as a high feed conversion ratio and low weight) through the horizontal transmission of this virus remain a challenge in adult chickens, with severe consequences on health and welfare [3,4,5].

CAV is a relatively small virus with a diameter of 23 nm [6]. It is a non-enveloped, icosahedral, single-stranded DNA virus that is grouped into the genus *Gyrovirus* and the family *Anelloviridae* [7]. It consists of three overlapping open reading frames encoding three viral proteins (VPs). VP1 which is the main structural capsid protein, is known to be antigenic, and can induce neutralizing antibodies in hosts [8]. The non-structural protein (VP2), which is involved in phosphatase activity, also functions as a scaffold, which helps in the correct assemblage of VP1 [9]. The co-expression of VP1 and VP2 has been reported to induce virus-neutralizing antibodies in chicken hosts, and as such, they have been regarded as immunogenic and potential vaccine candidates [6,10,11]. VP3, also known as apoptin, causes apoptosis, which leads to the depletion of lymphocytes by CAV [12].

The primary targets of CAV include the hemocytoblast of the bone marrow and the precursor lymphocytes of the thymus. The depletion of the hemocytoblast cells leads to a decrease in erythrocytes, granulocytes, and thrombocytes, which causes severe anemia, immunosuppression, and ultimately increases the susceptibility of the host to other secondary infections [2]. CAV infection progressively destroys precursor T-lymphocytes, which leads to a drastic depletion of the CD8^+^/CD4^+^ T-cell in the infected chicks [13].

Current live attenuated and inactivated vaccines against CAV disease have shown complete protection against vertical transmission of the virus that causes severe immunosuppressive symptoms, but the drawback of these vaccines, including the virulence reversion of the virus and the inability of the CAV strain to grow to high titer levels in an embryo or cell culture, constitute a challenge to vaccine development [14,15,16]. To circumvent these limitations, different experimental studies have reported the efficacy of DNA and recombinant vaccines in inducing high specific CAV antibody titers in vaccinated chickens [11,17,18]. Despite these advances, these vaccines are yet to be approved for use in chickens, which therefore means alternative strategies are needed for the design of a safe and effective vaccine against CAV disease.

Epitope-based vaccines derived through the immunoinformatics approach have received wide recognition in the design of novel vaccines against different pathogens [19,20,21]. The potential advantages of this vaccine vis-à-vis cost-effectiveness and the ability to induce both humoral and cellular immunity make it a suitable alternative vaccine for the control of CAV infection. The vaccination of breeder flocks with this kind of vaccine could provide their progeny with better immunity (maternally derived antibody) against clinical and sub-clinical infection of CAV. This study, therefore, designed a multiepitope vaccine consisting of T- and B-cell epitopes of combined CAV viral proteins VP1 and VP2.

## 2. Materials and Methods

### 2.1. Immunoinformatics of Viral Proteins

#### 2.1.1. Retrieval and Filtering of VP1 and VP2 Protein Sequences

A total of 1164 and 532 protein sequences of VP1 and VP2, respectively, were downloaded from NCBI database (https://www.ncbi.nlm.nih.gov/protein; accessed on 10 October 2021). The accession numbers of these sequences are reported in Supplementary Files S1 and S2. The multiple sequence alignment of the sequences was carried out with CLUSTALW server (https://www.genome.jp/tools-bin/clustalw, accessed on 10 October 2021). The conserved regions with a minimum of 15 amino acids were selected for antigenicity test with a threshold value of greater than or equal to 0.4 (≥0.4) using the Vaxijen v2.0 server [22]. The selected sequences that met antigenicity criteria were further screened for outer membrane test with TMHMM v2.0 server (http://www.cbs.dtu.dk/services/TMHMM/, accessed on 15 October 2021) using the default parameters.

#### 2.1.2. CD8^+^ T-Cell Epitopes and MHC-I Binding Allele Prediction

Due to the lack of chicken MHC alleles in immunoinformatics database, human HLA alleles have been used as substitute in most studies to predict T-cell epitopes in chickens [23,24]. Additionally, B-F alleles in chicken have been shown to be similar to human MHC-I alleles biochemically and functionally in antigen presentation and induction of immune response [25]. As such, the conserved sequences of VP1 and VP2 protein were subjected to the default parameters of NetCTL v1.2 server [26] for nonamers prediction. The generated nonamers with threshold values above 0.05 were used for the prediction of frequently and non-frequently major histocompatibility complex class I binding alleles (MHC-I) using IEDB server (http://tools.iedb.org/mhci/, accessed on 17 October 2021) with the following parameters: amino acid: 9, IC_50_: <250, and prediction method: stabilized matrix-based method (SMM).

#### 2.1.3. CD4^+^ T-Cell Epitopes and MHC-II Binding Allele Prediction

The same conserved sequences of VP1 and VP2 protein sequences were also used for the prediction of CD4^+^ T-cell epitopes and their binding alleles using IEDB MHC-II server (http://tools.iedb.org/mhcii/, accessed on 18 October 2021). The parameters used for the selection were amino acid length: 15; prediction method: SMM-aligned; IC_50_: <250.

#### 2.1.4. Immunogenicity, Antigenicity, Conservancy, and Allergenicity of Predicted Epitopes

The immunogenicity of the predicted CD8^+^ T-cell epitopes was determined with the default parameters of IEDB immunogenicity server (http://tools.iedb.org/immunogenicity/, accessed on 20 October 2021). The antigenicity of both the predicted CD8^+^ T-cell and CD4^+^ T-cell epitopes was also evaluated with Vaxijen v1.2 server with threshold values of greater than or equal to 0.5 (≥0.5) [22]. The predicted CD4^+^ T-cell epitopes were screened for the cytokines-inducing abilities-IFN-gamma [27] and IL-10 [28] with default parameters. Following this, the selected epitopes were screened for allergenicity [29], toxicity [30], and conservancy (https://tools.iedb.org/conservancy/, accessed on 20 October 2021) with CD8^+^ T-cell epitopes.

#### 2.1.5. Prediction of Linear B-Cell Epitopes

ABCpred server [31] was used for the prediction of octadecameric linear B-cell epitopes using the default parameters. The predicted B-cell epitopes were further screened for allergenicity, toxicity, and conservancy with CD8^+^ T-cell epitopes.

### 2.2. Multiepitope Vaccine Construction

#### 2.2.1. Multiepitope Vaccine (MEV) Design

The MEV was constructed by joining CD8^+^ T-cell, CD4^+^ T-cell epitopes, and B-cell epitopes. These selected epitopes are highly antigenic, non-allergen, and showed 100% conservancy, and as such, they meet the criteria for potential vaccine construction. The AAY linker fused the CD8^+^ T-cell epitopes while GPGPG and KK linkers fused CD4^+^ T-cell and B-cell epitopes, respectively. An adjuvant, human β-defensin 3 (Accession number: 1KJ6_A), which is a basic 45 amino-acid-long peptide with antimicrobial and immunomodulatory properties, was joined to the N-terminal end of CD8^+^ T-cell epitopes with the help of EAAAK linker. The MEV construct was screened for antigenicity and allergenicity.

#### 2.2.2. Physicochemical Properties and Secondary Structure Prediction

Protparam server (https://web.expasy.org/protparam/, accessed on 23 October 2021) was used to calculate physicochemical parameters of the MEV construct, which include the following: instability index, aliphatic index, theoretical isoelectric point (PI), half-life, and grand average hydropathy (GRAVY). The prediction of the secondary structure was also completed using the PSIPRED v4.0 server (http://bioinf.cs.ucl.ac.uk/psipred/, accessed on 23 October 2021).

#### 2.2.3. Prediction of Tertiary Structure, Refinement, and Validation

The 3D structure of the MEV construct was predicted with the trRosetta server [32], while the refinement was completed with Galaxy Refine Server [33]. The validation of the predicted 3D model was completed with ProSA-web and PROCHECK, where Z-score and Ramachandran analysis were calculated, respectively [34].

#### 2.2.4. Conformational B-Cell Epitopes Prediction

Conformational B-cell epitopes were predicted from the refined 3D structure of the MEV construct using ElliPro server [35]. The server has the best AUC score of 0.732 and is highly reliable in identifying antibody epitopes in protein antigens compared to other conformational B-cell epitope prediction tools [35].

#### 2.2.5. Molecular Docking of MEV with TLR3

Due to the involvement of TLR3 in antiviral response and innate immunity in both chickens and mammals (Karpala et al., 2008), TLR3 with pdb identifier (1ZIW) was retrieved in PDB format from the RCSB Protein Data Bank. Naccess tool was used to determine the active residues of the TLR3 that were used in docking with MEV with ATTRACT software. PDBsum online tool [36] was used to evaluate the protein–protein interaction of the TLR3 active residues and the MEV. The MEV construct carries the antigens of CAV parasite, and as such, the rationale for the molecular docking of MEV-TLR3 is to examine whether MEV will interact with TLR3 in chickens by actively binding to the active site of the TLR3.

#### 2.2.6. Molecular Dynamics of the MEV Construct

The stability of the MEV-TLR complex was carried out with iMODS server [37]. The server evaluates protein stability by using normal mode analysis (NMA) to generate the internal coordinates of the protein. The following parameters vis-à-vis deformability plot, eigenvalue, B-factor value, covariance matrix, and elastic network model were used to describe the stability of the MEV construct.

#### 2.2.7. Reverse Translation, Codon Optimization, and In Silico Cloning of the MEV

Java Codon Adaptation Tool (JCAT) server (http://www.jcat.de/, accessed on 25 October 2021) was used for codon optimization and reverse translation. This generated cDNA of the vaccine that can be expressed in *E. coli* K-12 strain. The codon adaptation index (CAI) and GC content were also calculated using the JCAT server. The sticky end restriction sites of *SacI* and *BamHI* were added to both the N-terminal (start) and C-terminal (end) of the optimized sequence. The optimized MEV sequences were inserted into the multiple cloning sites (MCS) of pET-28a (+) plasmid using SnapGene tool (https://www.snapgene.com/, accessed on 25 October 2021).

#### 2.2.8. Immune Simulation of MEV Construct

C-ImmSim 10.1 server (http://kraken.iac.rm.cnr.it/C-IMMSIM/, accessed on 30 October 2021) was used to predict the antibody-inducing abilities of the MEV construct. The abilities of the MEV construct to induce immune cells, such as B-cells, NK cells, dendritic cells, HTL, CTL, immunoglobulins, and cytokines, were simulated by this server. The minimum recommended time between dose 1 and dose 2 of the most commonly used vaccine is 4 weeks. As such, two vaccine injections containing a thousand units of MEV were administered in the C-ImmSim server in the following orders: dose 1 (1 time-step (equivalence of 8 h of real-life) and dose 2 (84 time-steps) for a total of 1000 steps simulation.

## 3. Results

### 3.1. Sequence Alignment, Antigenicity, and Membrane Test

The multiple sequence alignment analysis of the 1164 and 532 protein sequences of VP1 and VP2 generated 180 and 190 conserved sequences, which were later reduced to 66 and 50 sequences, respectively, after satisfying antigenicity and outer membrane test criteria (Supplementary Files S3 and S4).

### 3.2. CD8^+^ T-Cell Epitopes and MHC-I Binding Allele Prediction

The conserved sequences for both VP1 and VP2 that met antigenicity and membrane criteria were used in the prediction of nonamers using the NetCTL server with a threshold of 0.05. This generated 645 and 636 nonamers for both VP1 and VP2, respectively, which was later used for the prediction of potential CD8^+^ T-cell epitopes using the IEDB MHC-I server with the parameter of an IC_50_ less than 250. A total of 159 and 131 potential CD8^+^ T-cell epitopes were generated for both VP1 and VP2, which were later reduced to 40 CD8+ T-cell epitopes for both viral proteins after they were successfully screened for antigenicity and immunogenicity. The selected CD8^+^ T-cell epitopes also overlapped with cytokines-inducing CD4^+^ T-cell epitopes and were non-allergenic (Table 1). Among the generated CD8^+^ T-cell epitopes, TMTIRFQGL and LTEGLLLPK had the highest and lowest antigenicity scores of 1.1407 and 0.5185, whilst the highest and lowest immunogenicity scores of 0.4584 and 0.0295 were found in DPDWYRWNY and LTEGLLLPK epitopes, respectively. The epitope ‘LMTIRFQGV’ is bound to the highest number of MHC-I binding alleles (Table 1). All the generated epitopes in Table 1 were conserved, non-allergenic, and IFN-gamma and IL-4 inducers.

### 3.3. CD4^+^ T-Cell Epitopes and MHC-II Binding Allele Prediction, Conservancy, and Allergenicity

The prediction of CD4^+^ T-cell epitopes based on the 66 and 50 conserved sequences of both viral proteins was completed using the IEDB MCH-II server with IC_50_ less than 250 and the SMM-align method set as criteria. This generated 556 and 126 potential CD4^+^ T-cell epitopes, which were later reduced to 316 and 72 epitopes, respectively, after screening for their antigenicity. These potential CD4^+^ T-cell epitopes, which overlapped with CD8^+^ T-cell epitopes, were also screened for their cytokine (IFN-gamma and IL-10)-inducing abilities followed by an allergenicity and toxicity test. This reduced the potential CD4^+^ T-cells epitopes to 11 and 3 for both viral proteins, respectively (Table 2). Of the final generated epitopes for both viral proteins, the highest and lowest antigenicity scores of 0.8821 and 0.5000 were found in QSTMTIRFQGLIFLT and FRKAFHNPRPGAYSV epitopes, respectively. The highest MHC-II binding alleles of six were bound to the LLMTIRFQGVIFLTE epitope (Table 2). The generated epitopes were all conserved, non-toxic, non-allergenic, and were inducers of IFN-gamma and IL-4.

### 3.4. Linear B-Cell Epitopes Prediction

The conserved sequences of both viral proteins generated 217 and 167 linear B-cell epitopes, respectively, using the ABCpred server. Further screening of these predicted B-cell epitopes for antigenicity reduced them to 141 and 104 sequences. After the subsequent screening of these antigenic linear B-cell epitopes for allergenicity, toxicity, and conservancy with CD8^+^ T-cell epitopes, they were reduced to 12 and 1 potential B-cell epitopes in both viral proteins, respectively (Table 3).

### 3.5. MEV Construction and Validation

#### 3.5.1. MEV Construction and Screening

**Figure 1:**
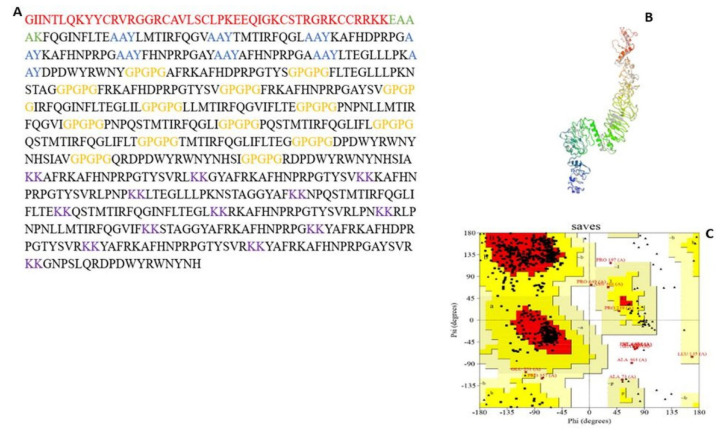
The 3D structure prediction and validation of MEVconstruct. (**A**) MEV construct sequence with epitopes in black; adjuvant in red; and linkers in blue, orange, green and purple. (**B**) Refined model of MEV construct. (**C**) Ramachandran analysis plot with 84.4% in most favored region.

As shown in Figure 1, the constructed MEV consists of 9 CD8^+^ T-cell, 14 CD4^+^ T-cell, and 13 B-cell epitopes joined together by AAY, GPGPG, and KK linkers, respectively. To boost the immunogenic property of the MEV, Human β-defensin 3 was attached as an adjuvant to the N-terminal of the constructed MEV through the EAAK linker. Further screening of the MEV construct through Vaxijen and AllerTop servers confirmed the vaccine to be antigenic (antigenic score: 0.6055) and non-allergenic.

#### 3.5.2. Physicochemical Properties and Secondary Structure Prediction

**Figure 2:**
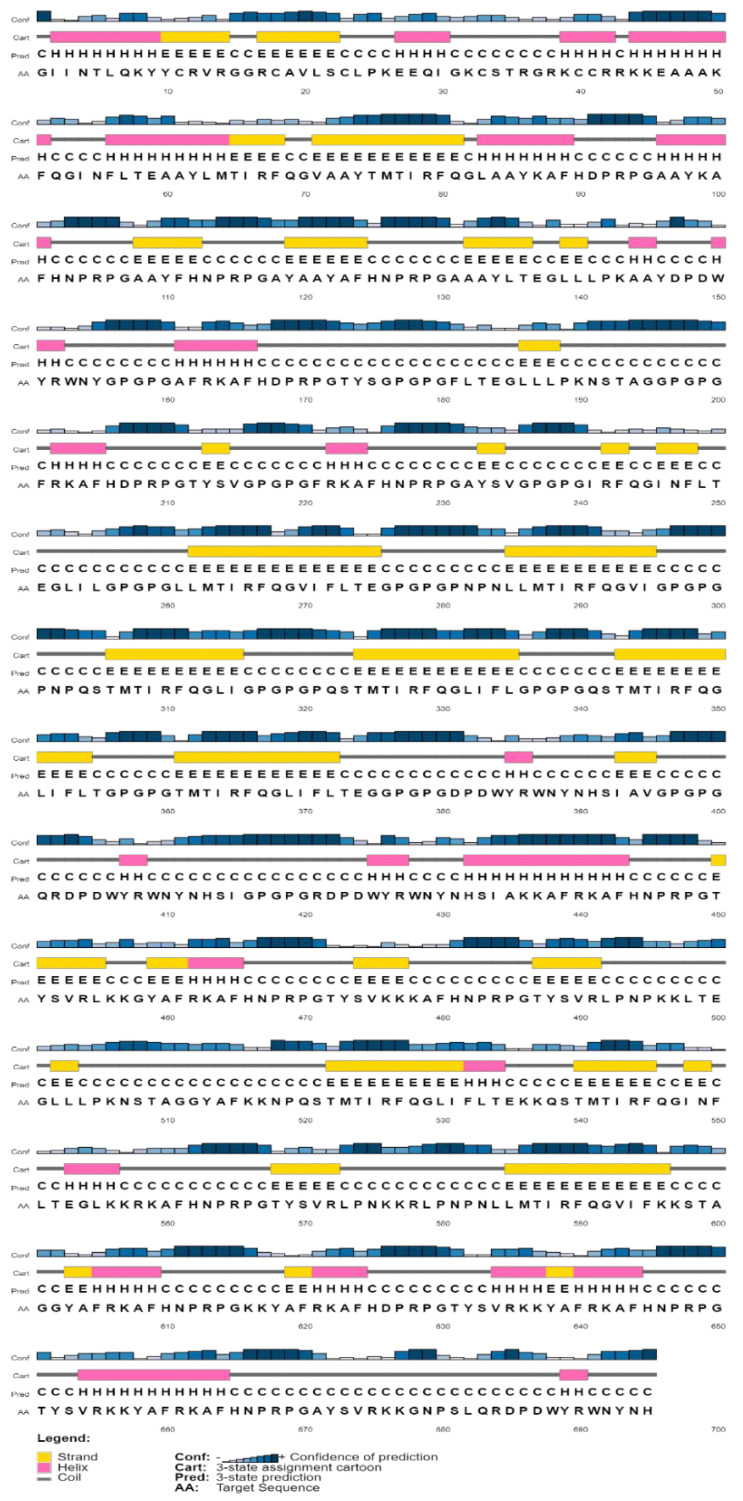
Secondary structure prediction of the MEV construct.

Protparam server showed the physicochemical properties of MEV under the following parameters: molecular weight of 77.6 kDa; a theoretical isoelectric (pI) value of 10.72, indicating the basic nature of the MEV; instability and aliphatic index of 30.21 and 55.54, respectively, also pointing to the stability of the vaccine; and a grand average of hydropathicity (GRAVY) value of −0.637, also showing the hydrophilic nature of the vaccine. Similarly, an estimated half-life of greater than 20 h and 10 h were detected for the in vivo analysis of the constructed MEV in both yeast and *E. coli*, respectively. The secondary structure was dominated by a coil structure (378/695; 54.38%), alpha-helix (125/695; 17.98%), and strand (192/695; 27.63%), as shown in Figure 2.

#### 3.5.3. Tertiary Structure Prediction, Refinement, and Validation

The prediction of the MEV 3D structure was completed with the trRosetta server (Figure 1). The validation of the predicted 3D structure with PROCHECK shows a Ramachandran plot of 79.7% in the most favored region. This was further subjected to refinement with the Galaxy Refine server, with model 1 selected as the best model, as indicated by the following parameters: GDT-HA (0.9529), RMSD (0.410), MolProbity (2.337), clash score (21.6), poor rotamers (0.6), and Ramachandran plot (91.3). The validation of this refined model with PROCHECK showed an improvement in the quality of the model with a Ramachandran plot of 84.4% in the most favored region, 13.9% and 0.8% in additional and generously allowed regions, while 0.9% of the residues were in disallowed regions, respectively (Figure 1). The MEV construct also falls within the range of native proteins with a Z score of −10.08 using the ProSAweb server. 

#### 3.5.4. Conformational B-Cell Epitopes Prediction

**Figure 3:**
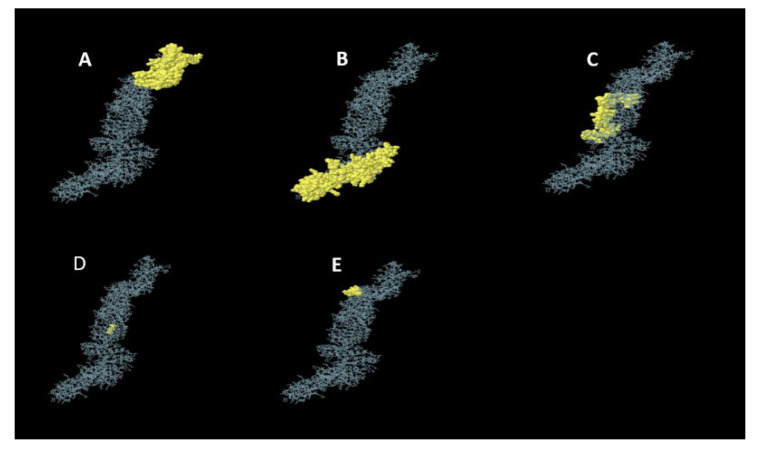
The 3D structure of the 5 predicted conformational (discontinuous) B-cell epitopes in the refined MEV construct. The yellow parts are the conformational B-cell epitopes, while the gray parts are the rest of the residues. (**A**) A total of 161 residues with a score of 0.774. (**B**) A total of 183 residues with score of 0.696. (**C**) A total of 76 residues with a score of 0.630. (**D**) A total of 3 residues with a score of 0.547. (**E**) A total of 7 residues with a score of 0.543.

ElliPro server with default parameters predicted five conformational B-cell epitopes from the refined 3D model of the MEV construct. These conformational B-cell epitopes consist of 430 residues with a score ranging from 0.543 to 0.774. The details of the 3D constructions of these epitopes are shown in Figure 3.

#### 3.5.5. Interaction Analysis of MEV Construct and TLR-3

**Figure 4:**
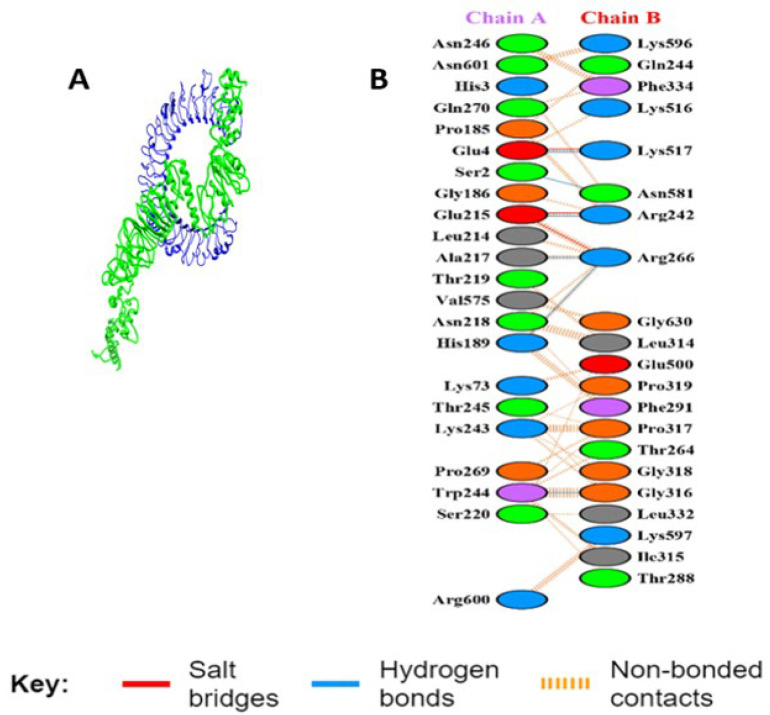
The feature showing (**A**) the docked complex of MEV (green) and TLR3 (blue) and (**B**) the residue interactions of TLR3 (chain A) and MEV (chain B).

The refined 3D structure of the MEV construct was docked with the 3D construction of TLR3 using the online ATTRACT software. The strong interaction of the MEV-TLR3 was evaluated by their low binding energy (−300.755 kcal/mol). The binding interaction of the MEV and the TLR3 residues is also shown in Figure 4, where MEV formed six hydrogen bonds with the following TLR3 residues: Ser 2, Glu 4, Glu 215, Ala 217, Asn 219, and Trp 244.

#### 3.5.6. Molecular Dynamics Simulation of the MEV Construct

**Figure 5:**
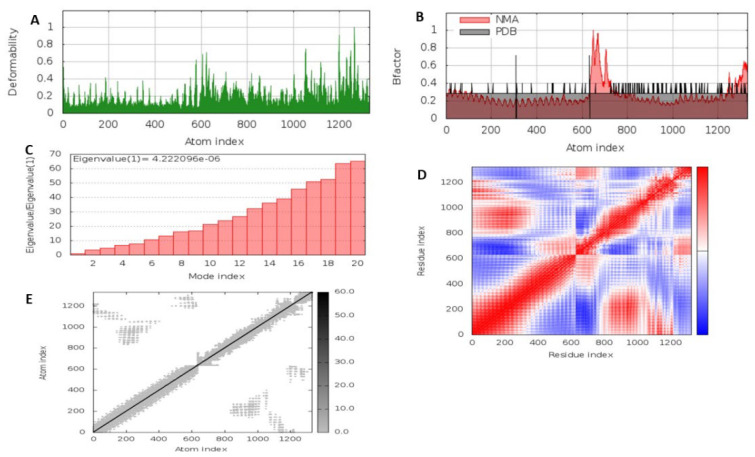
Molecular dynamics with iMODS server. (**A**) Deformability plot. (**B**) B factor plot. (**C**) The eigenvalue of the MEV-TLR complex. (**D**) The covariance matrix between pairs of residues where red, white, and blue represent correlated, uncorrelated, and anti-correlated motion, respectively. (**E**) The elastic network model, with darker gray suggesting more rigid springs.

Molecular dynamics using the iMODS server was used to evaluate the stability and movement of the MEV-TLR complex. As shown in Figure 5A, the deformability analysis shows that there was minimal distortion in the complex, as indicated by the hinges. This was complemented by the B-factor plot, which is proportional to the root mean square (RMS) and shows the stability of the docked MEV-TLR complex (Figure 5B). Additionally, a higher eigenvalue of 4.222 × 10^−6^ was observed, which shows the energy required to deform the MEV-TLR complex, and as such, suggests its stability (Figure 5C). The covariance matrix between the pairs of residues is indicated in the graph, as shown in Figure 5D, where red, white, and blue represent correlated, uncorrelated, and anti-correlated motion, respectively. The elastic network model of the MEV-TLR complex (Figure 5E) studies the stiffness of the complex. A darker gray means a higher protein stiffness in certain regions.

#### 3.5.7. Reverse Translation, Codon Optimization, and In Silico Cloning

**Figure 6:**
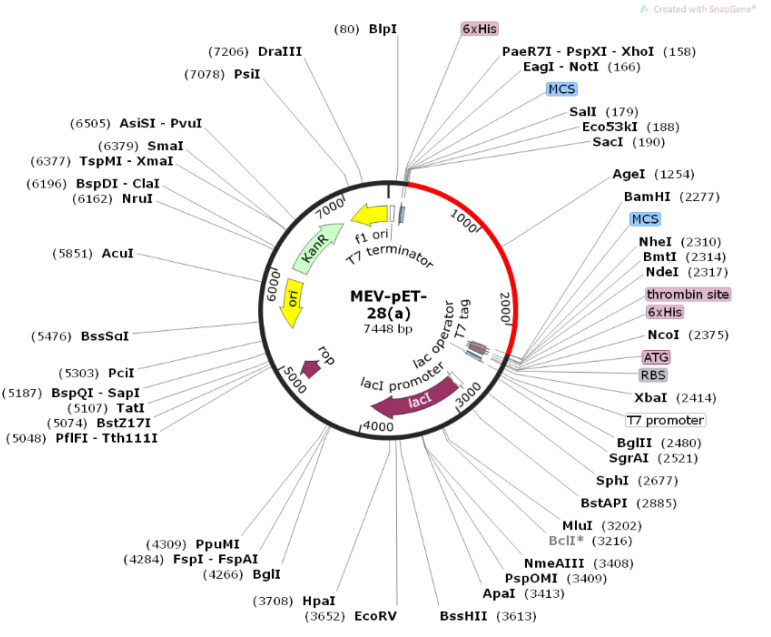
In silico cloning of the MEV construct into pET-28a (+) vector. The insert is shown in red.

The codon optimization of the vaccine construct using JCAT generated a cDNA, which is 2085 nucleotides long with a CAI score value and GC content of 1.0 and 55.5%, respectively. Additionally, the sticky end restriction sites of *SacI* and *BamHI* were added to both the N-terminal (start) and C-terminal (end) of the optimized sequence, which was later cloned in silico in the multiple cloning site (MCS) of the pET-28a (+) cloning vector using the SnapGene tool (Figure 6).

#### 3.5.8. Immune Simulation

**Figure 7:**
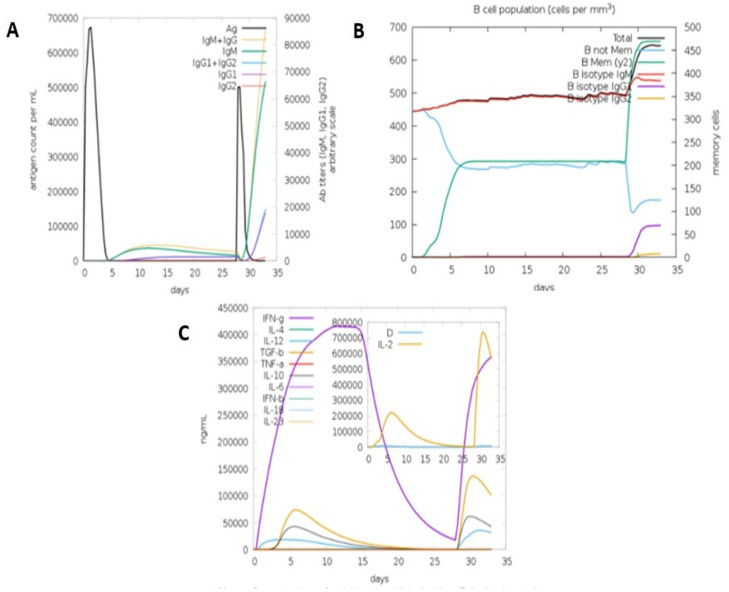
The (**A**) the induction of antibody responses after 2 doses of MEV vaccine. (**B**) The induction of B-cell population. (**C**) The expression of cytokines following MEV vaccine administration.

Initial induction of the primary antibody response (IgM), as shown in Figure 7, followed by high-level expression of both secondary and tertiary responses (IgM + IgG and IgG1 + IgG2) was predicted by the C-ImmSim simulation for the MEV construct. The administration of the MEV vaccine was also predicted to induce memory B-cell, which is important for the host’s protection against viral reinfection. Cytokine expressions, such IFN-gamma, TGF-b, IL-10, and IL-12, were also predicted, and this is consistent with the IFN-gamma- and IL-10-inducing abilities of our predicted epitopes, as indicated by IFNepitope and IL-10 Pred servers.

## 4. Discussion

The severity of the clinical and sub-clinical symptoms of CAV disease causes global morbidity, mortality, and economic losses in the poultry industry [17]. The constraint of the currently available vaccines has not only heightened the concern for this viral disease but has also necessitated the need to explore other alternative strategies to design a vaccine against CAV.

This present study reported IFN-γ and IL-10-inducer CD4^+^ and CD8^+^ T-cell epitopes that could induce a cellular immune response against CAV infection. Although clinical infection of the virus has been shown to drastically deplete CD4^+^ and CD8^+^ within the first 21 dpi [38] their inclusion in vaccine design is essential to induce lasting humoral responses. Transcriptomic analysis of cytokine expression during CAV infection also indicated the upregulation of IFN-γ and IL-10 at 7 and 11 dpi in the thymus and bone marrow [1,39]. IFN-γ activates macrophages, enhances the activity of Th1 cells, and as such, promotes innate and adaptive responses in response to infection in the host [40]. IL-10 is an anti-inflammatory cytokine that protects the host against tissue damage during the acute phase of the immune response [41].

Antibody production has been reported by different studies to provide complete protection against severe immunosuppressive symptoms of CAV [11,17,42]. B-cell epitopes that are antigenic and non-allergenic were also predicted in this study. These epitopes were validated by the immune simulation software, which predicted the induction of primary (IgM), secondary (IgM + IgG), and tertiary (IgG1 + IgG2) antibody responses against CAV infection. Memory B-cell was also predicted, which is important for the protection against reinfection of the virus.

Multiepitope consisting of T-cell and B-cell epitopes were constructed with GPGPG, AAY, KK, and EAAAK linkers with β-defensin as an adjuvant. The linkers and adjuvant contribute to the potency and immunogenicity of the MEV construct, respectively [43]. The MEV construct was also predicted to be antigenic and non-allergenic, which suggests its safety and ability to induce an immune response against CAV. Different studies have used this same approach to construct a multiepitope vaccine against different pathogens [19,44,45]. The interaction of the MEV construct with TLR3 was also evaluated by molecular docking, which generated the lowest binding energy. TLR3 is known to induce an antiviral response and innate immunity signaling pathways [46]. The MEV construct was also predicted to be thermally stable and basic, with an aliphatic index and theoretical point of 55.54 and 10.72, respectively. A GRAVY score of −0.637 indicates the hydrophilic nature of the MEV construct. A Ramachandran plot with 84.4% of residues in the most favored regions, 13.9% and 0.8% in additional and generously allowed regions, and 0.9% of the residues in the disallowed region suggests the quality of the MEV 3D model.

The molecular dynamics of the MEV-TLR complex, which was carried out with the iMODS server, confirmed the stability of the MEV construct, as indicated by the minimal distortion of the deformability plot supported by high eigenvalues. To avoid mRNA codon inconsistency, which could lead to variation in foreign gene expression in the host cell genome, the MEV construct was codon-optimized for potential expression in *E. coli* K12. This was later cloned into vector-pET+28a (−).

## 5. Conclusions

Vaccination has been the only safe and effective control measure used to curtail the severity of the immunosuppressive symptoms caused by the vertical transmission of CAV in young chicks less than 3 weeks of age. Breeder flocks, when vaccinated with the currently available live attenuated and inactivated vaccines, transfer maternally derived antibodies to their progeny. However, the shortcomings of these vaccines, which include the virulent reversion of CAV strain and the inability to achieve high titer levels of the CAV strain when grown in the embryo and cell culture, have necessitated a search for a novel and effective vaccine against CAV. The multiepitope vaccine presented in this study is highly immunogenic, antigenic, non-allergen, and capable of inducing strong cellular and humoral immune response when used in breeder flocks. It is believed that this vaccine will produce adequate maternal antibodies that can protect young chicks against the clinical symptoms of CAV infection. Although the proposed vaccine in this study is based on in silico prediction and requires further experimental validations, it serves as a very important step towards designing a potential vaccine.

## Figures and Tables

**Table 1:** The list of selected CD8^+^ T-cells epitopes of VP1 and VP2 interacting with MHC class I HLA-alleles.

Viral Protein	CD8+ T-Cell Epitopes	Antigenicity Score	Immunogenicity	MHCI-Alleles
VP1	FQGINFLTE	0.5863	0.2621	HLA-A*02:06
	LMTIRFQGV	1.0143	0.2292	HLA-A*02:06, HLA-A*02:03, HLA-B*08:01, HLA-A*02:01
	TMTIRFQGL	1.1407	0.2292	HLA-B*08:01
	KAFHDPRPG	0.7871	0.1189	HLA-A*30:01
	KAFHNPRPG	0.5687	0.0910	HLA-A*30:01
	FHNPRPGAY	0.6146	0.0782	HLA-A*30:02, HLA-B*35:01
	AFHNPRPGA	0.5667	0.0524	HLA-A*30:01
	LTEGLLLPK	0.5185	0.0295	HLA-A*11:01
VP2	DPDWYRWNY	0.9093	0.4584	HLA-B*35:01

**Table 2:** The list of selected CD4^+^ T-cells epitopes of VP1 and VP2 interacting with MHC class II HLA-alleles.

Viral Protein	CD4+ T-Cell Epitopes	Antigenicity Score	MHCII-Alleles
VP1	AFRKAFHDPRPGTYS	0.5598	HLA-DRB5*01:01
	FLTEGLLLPKNSTAG	0.5757	HLA-DRB1*01:01, HLA-DPA1*03:01
	FRKAFHDPRPGTYSV	0.6011	HLA-DRB5*01:01
	FRKAFHNPRPGAYSV	0.5000	HLA-DRB5*01:01
	IRFQGINFLTEGLIL	0.7592	HLA-DPA1*01:03, HLA-DPA1*03:01, HLA-DPA1*02:01
	LLMTIRFQGVIFLTE	0.8222	HLA-DRB1*04:05, HLA-DRB1*01:01, HLA-DRB1*15:01, HLA-DPA1*02:01, HLA-DPA1*03:01, HLA-DPA1*01:03
	PNPNLLMTIRFQGVI	0.8698	HLA-DRB4*01:01, HLA-DPA1*03:01, HLA-DPA1*01:03, HLA-DRB1*11: 01, HLA-DRB1*01:01
	PNPQSTMTIRFQGLI	0.8712	HLA-DRB4*01:01
	PQSTMTIRFQGLIFL	0.7590	HLA-DRB1*15:01, HLA-DRB4*01:01, HLA-DPA1*02:01, HLA-DPA1*03:01
	QSTMTIRFQGLIFLT	0.8821	HLA-DRB1*15:01, HLA-DRB4*01:01, HLA-DPA1*02:01, HLA-DPA1*03:01
	TMTIRFQGLIFLTEG	0.8422	HLA-DRB1*04:05, HLA-DRB1*15:01, HLA-DRB4*01:01, HLA-DPA1*02:01, HLA-DPA1*03:01
VP2	DPDWYRWNYNHSIAV	0.7586	HLA-DRB1*13:02, HLA-DRB3*01:01
	QRDPDWYRWNYNHSI	0.8368	HLA-DRB1*07:01, HLA-DRB3*01:01
	RDPDWYRWNYNHSIA	0.8539	HLA-DRB1*07:01, HLA-DRB3*01:01

**Table 3:** Predicted linear B-cell epitopes showing their antigenicity.

Viral Protein	B-Cell Epitopes	Antigenicity Score	ABCpred Score
VP1	AFRKAFHNPRPGTYSVRL	0.6040	0.84
	GYAFRKAFHNPRPGTYSV	0.5284	0.71
	KAFHNPRPGTYSVRLPNP	0.6401	0.89
	LTEGLLLPKNSTAGGYAF	0.6674	0.77
	NPQSTMTIRFQGLIFLTE	0.8296	0.65
	QSTMTIRFQGINFLTEGL	0.8772	0.77
	RKAFHNPRPGTYSVRLPN	0.7101	0.86
	RLPNPNLLMTIRFQGVIF	0.5641	0.74
	STAGGYAFRKAFHNPRPG	0.6224	0.65
	YAFRKAFHDPRPGTYSVR	0.6751	0.74
	YAFRKAFHNPRPGAYSVR	0.8513	0.78
	YAFRKAFHNPRPGTYSVR	0.7547	0.88
VP2	GNPSLQRDPDWYRWNYNH	0.5966	0.80

## Data Availability

Not applicable.

## Supplementary Materials

The following supporting information can be downloaded at: https://www.mdpi.com/article/10.3390/v14071456/s1, File S1: List of CAV VP1 protein sequences; File S2: List of CAV VP2 protein sequences; File S3: List of 66 VP1 protein sequences that satisfied antigenic and membrane test criteria; File S4: List of 50 VP2 protein sequences that satisfied antigenic and membrane test criteria.
